# Prenatal Diagnosis of Fetal Obstructed Hemivagina and Ipsilateral Renal Agenesis (OHVIRA) Syndrome

**DOI:** 10.3390/medicina59040703

**Published:** 2023-04-04

**Authors:** Soo Jung Kim, So-Yeon Shim, Hyun-Hae Cho, Mi-Hye Park, Kyung A. Lee

**Affiliations:** 1 Department of Obstetrics and Gynecology, Ewha Womans University College of Medicine, Ewha Womans University Seoul Hospital, Seoul 07804, Republic of Korea; bossksj25@gmail.com (S.J.K.);; 2 Department of Pediatrics, Ewha Womans University College of Medicine, Ewha Womans University Seoul Hospital, Seoul 07804, Republic of Korea; 3 Department of Radiology, Ewha Womans University College of Medicine, Ewha Womans University Seoul Hospital, Seoul 07804, Republic of Korea

**Keywords:** Herlyn-Werner-Wunderlich syndrome, obstructed hemivagina and ipsilateral renal agenesis syndrome (OHVIRA), Gartner duct cyst, prenatal diagnosis

## Abstract

*Background*: Obstructed hemivagina and ipsilateral renal agenesis (OHVIRA) syndrome, also known as Herlyn-Werner-Wunderlich syndrome, is a rare syndrome characterized by the triad of uterus didelphys, obstructed hemivagina, and ipsilateral renal agenesis. Most cases of OHVIRA have been reported in adolescents or adults. Gartner duct cysts, including those manifesting as vaginal wall cysts, are also rare. Fetal OHVIRA syndrome and Gartner duct cysts are difficult to diagnose. *Case Presentation*: Here, the authors report a case of combined OHVIRA and Gartner duct cyst diagnosed prenatally by ultrasonography, along with a brief review of the relevant published reports. A 30-year-old nulliparous female was referred to our institution at 32 weeks’ gestation for fetal right kidney agenesis. Detailed ultrasonographic examinations using 2D, 3D, and Doppler ultrasounds revealed hydrocolpometra, and uterus didelphys, with a normal anus and right kidney agenesis. *Conclusions*: When encountering female fetuses with ipsilateral renal agenesis or vaginal cysts, clinicians should be aware of OHVIRA syndrome and Gartner duct cysts and perform systematic ultrasonographic examinations for other genitourinary anomalies.

## 1. Introduction

Obstructed hemivagina and ipsilateral renal agenesis (OHVIRA) syndrome, also known as Herlyn-Werner-Wunderlich syndrome, is a rare syndrome characterized by the triad of uterus didelphys, obstructed hemivagina, and ipsilateral renal agenesis. In 1922, Purslow first reported this syndrome [1]. In 1971, Herlyn and Werner reported a case of renal agenesis with a blind hemivagina, and Wunderlich described the association of renal aplasia, a bicornuate uterus with a simple vagina, and an isolated hematocervix in 1976 [2].

OHVIRA syndrome is believed to be related to the abnormal development of the Müllerian ducts during the eighth week of gestation, but the exact pathogenesis is unclear. It is a rare congenital abnormality of the genitourinary system with an estimated incidence of 0.1–3.8% [3]. Most cases have been reported in adolescents or adults after menarche and have included progressive dysmenorrhea, abnormal pain, menstrual irregularities, and pelvic masses. The clinical presentation of OHVIRA syndrome can vary significantly, making it essential to consider differential diagnoses for unilateral kidney agenesis in adolescent women, including obstructive genital tract anomalies and Gartner duct cysts. An accurate diagnosis is crucial to preventing potential complications such as endometriosis, infertility, and abortion. As a result, timely follow-up and treatment, particularly during adolescence, can aid in preventing these long-term consequences. It is difficult to diagnose fetal OHVIRA syndrome.

Gartner duct cysts occur as a result of obstruction of the mesonephric duct system during fetal development and represent cystic remnants [4,5]. These cysts can be associated with an abnormal genitourinary system, such as in the context of OHVIRA syndrome, and they may be located submucosally along the anterior or lateral wall of the vagina [6]. Accurate diagnosis of Gartner duct cysts requires a combination of physical examination and imaging modalities such as ultrasound and MRI. The management options for Gartner duct cysts include aspiration, deroofing, or complete cyst removal via a vaginal approach. The appropriate timing for treatment is not yet clear, particularly in the case of newborns.

Recently, fetal cases have been diagnosed thanks to the development and use of prenatal ultrasound examinations. Here, the authors report a case of OHVIRA diagnosed prenatally by ultrasound examinations, with Gartner duct cysts detected after birth. 

## 2. Case

A nulliparous woman, aged 30 years, was referred to our institution at 32 weeks of gestation for further investigation of fetal right kidney agenesis. The patient denied experiencing any symptoms and had no relevant family history or prior kidney issues. Prenatal screening tests, including the Sequential test, showed low risk, and the maternal oral glucose tolerance test was normal. Upon physical examination, no specific findings were noted, and the cervix was found to be closed.

**Figure 1:**
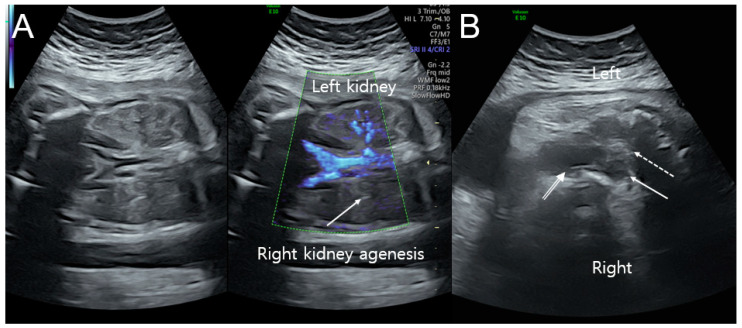
Ultrasonographic findings at prenatal examination. (**A**) Color Doppler ultrasound of the left kidney with the renal artery and right kidney agenesis (arrow) without the renal artery at 32 weeks of gestation. (**B**) Uterine didelphys with right hydrocolpometra (arrow), left uterus (dotted arrow), and vaginal cyst (double-lined arrow) at 37 weeks of gestation.

A transabdominal ultrasound scan showed a normally growing female fetus with right kidney agenesis. On a transabdominal ultrasound scan, the estimated fetal weight and abdominal circumference were consistent with 32 weeks of gestation, with adequate amniotic fluid and no placental abnormalities observed. The fetal brain and heart were normal, and Doppler waveforms from the umbilical artery and middle cerebral artery were within acceptable ranges. Additionally, detailed ultrasonographic examinations using 2D and Doppler ultrasounds revealed right kidney agenesis, uterine didelphys, hydrocolpometra, and a vaginal cyst (Figure 1A,B). Based on the imaging, fetal OHVIRA syndrome was highly suspected.

At 39 weeks and 3 days of gestation, the amniotic sac membrane ruptured; however, labor did not proceed, and an emergency cesarean delivery was performed at 39 weeks and 4 days of gestation. The patient delivered a healthy female newborn with a birth weight of 3190 g, a 1 min Apgar score of 8, and a 5 min Apgar score of 10. There were no problems during the postpartum period. 

After birth, the newborn was admitted to the neonatal intensive care unit for evaluation of the genitourinary system for any malformations. The newborn’s weight at birth, 3190 g, was appropriate for gestational age, and her height, head circumference, and chest circumference were 51.5 cm, 34 cm, and 33 cm, respectively. The neonate appeared healthy and active, with symmetric chest movements, normal breathing and respiratory patterns, and no evidence of pectus excavatum. On the second day after birth, an abdominal ultrasound revealed probable right kidney agenesis with underlying uterine didelphys, a small amount of fluid collected within the right side of the uterine fundus, and a fluid-filled dilated right hemivagina. Additionally, the abdominal ultrasound detected three simple cysts two days after birth.

**Figure 2:**
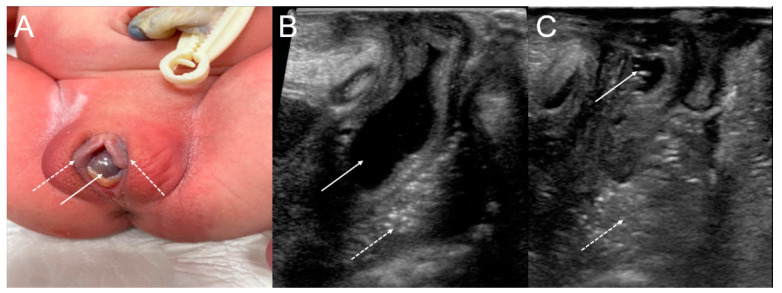
(**A**) The cystic bulging mass (arrow) arising from the right vaginal wall between the labia minora (dotted arrow) after birth. (**B**) On the initial ultrasound with a perineal approach, a fluid-filled cystic lesion (arrow) in the right pelvic cavity was noted anterior to the rectum (dotted arrow). (**C**) After aspiration, the previously observed cystic lesion collapsed (arrow).

Perineal inspection revealed a normal anus, a normally situated urethral meatus, a bulge at the vaginal introitus, and a cystic mass protruding between the labia minora (Figure 2A). The remainder of the physical examination was unremarkable. 

When the baby was eight days old, an ultrasound-guided needle aspiration of the cystic mass protruding between the labia minora, not the vaginal septum, was performed, and yellow serous fluid was aspirated. The collapsed cystic lesion was observed after the needle aspiration, and its appearance was suggestive of a Gartner duct cyst diagnosis (Figure 2B,C).

**Figure 3:**
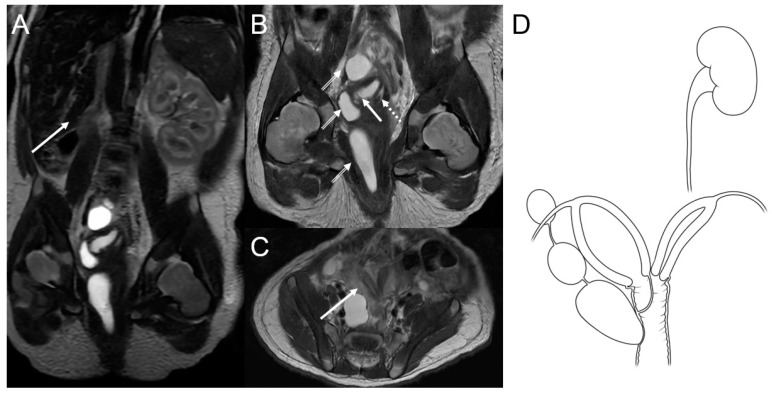
(**A**) Coronal T2-weighted abdominopelvic magnetic resonance image showing an absent right kidney (arrow) with compensatory left kidney hypertrophy. (**B**) Coronal T2-weighted image of the pelvic area showing uterine didelphys with an obstructed right hemivagina and hydrocolpometra (arrow) as well as the non-obstructed left hemivagina (dotted arrow). Three cystic lesions along the right hemivagina were thought to be tubulocystic anomalies, suggesting a diagnosis of Gartner duct cysts (double-lined arrows). (**C**) Axial T2-weighted images showing uterine didelphys (arrow). (**D**) Schematic diagram of OHVIRA syndrome and possible Gartner cysts.

A magnetic resonance imaging (MRI) scan of the baby’s abdomen and pelvis was done to see how three cystic masses were related to the vaginal wall. The MRI, performed on the fourth day after birth, revealed agenesis of the right kidney with a compensatory hypertrophied left kidney (Figure 3A), uterine didelphys, an obstructed right hemivagina with hydrocolpometra, a non-obstructed left hemivagina, and three cystic lesions along the right hemivagina. These cystic lesions were thought to be tubulocystic anomalies (Figure 3B,C). Therefore, the diagnosis of OHVIRA syndrome and Gartner duct cyst was established (Figure 3D). 

The newborn was discharged on the eleventh day after birth and examined 3 weeks later, and there were no problems. The child is undergoing follow-up at an outpatient clinic by a pediatric nephrologist.

## 3. Discussion

The true incidence of OHVIRA syndrome is unknown, but it is estimated to be between 0.1% and 3.8% [3]. Biological sex is established at fertilization, but the fetus begins to attain sexual characteristics by the seventh week of gestation. The genital system is closely related to the urinary system, and the development of these two systems is closely linked. The development of the Müllerian ducts—the embryonic formation of the female reproductive tract—includes elongation, fusion, canalization, and septal resorption. OHVIRA syndrome is thought to result from abnormal development of the Müllerian ducts during the fetal period, but the exact pathogenetic mechanism is unclear [3,7]. 

There are many classification systems for Müllerian abnormalities. The American Fertility Society (AFS) classification has been most widely used since 1988. Recently, however, new anatomic variants have been discovered, so a new classification was needed. The American Society for Reproductive Medicine (ASRM) updated the AFS’s 1988 classification with simple and descriptive terminology for identifying anomalies of the uterus, cervix, and vagina. The ASRM Müllerian Anomalies Classification 2021 (MAC 2021) classifies Müllerian anomalies into nine categories and provides guidance on diagnostic and treatment options. Anomalies are classified into different categories based on descriptive terminology, which include Müllerian agenesis, cervical agenesis, unicornuate uterus, uterus didelphys, bicornuate uterus, septate uterus, longitudinal vaginal septum, transverse vaginal septum, and complex anomalies [8].

The most common symptoms of OHVIRA syndrome are nonspecific, with patients complaining of lower abdominal and pelvic pain, progressive dysmenorrhea, or cystic masses in the vaginal wall after menarche [9]. Additionally, the obstructed vaginal septum affects the menstrual flow and causes urinary incontinence, endometriosis, and pelvic infections, such as abscesses, pyosalpinx, and peritonitis [10]. Endometriosis and subsequent pelvic adhesions may result in infertility or miscarriages [11]. The diagnosis of OHVIRA syndrome is often delayed until after the early reproductive years due to the normal external genitalia [12,13]. Moreover, ipsilateral anomalies of the urinary system, such as kidney agenesis, dysplastic or polycystic kidney, and ectopic or duplicated ureters, have been reported. Coexisting urologic anomalies can result in recurrent urinary tract infections [10].

Early diagnosis of OHVIRA syndrome is important for managing clinical symptoms and preventing complications. A speculum examination may reveal features suggestive of OHVIRA syndrome, but radiologic examinations are essential for the definitive diagnosis [12]. MRI is the gold standard for imaging and identifying Müllerian anomalies, as it provides details about uterine morphology and vaginal luminal continuity. MRI can also identify renal abnormalities [14]. Additionally, 3D transvaginal ultrasound and 3D computed tomography can provide accurate anatomical information about the uterine cavity’s external contour and internal shape [10]. These results can help determine appropriate treatment methods. 

OHVIRA is typically diagnosed during the peri-pubertal period after the manifestation of menstruation-related problems, including dysmenorrhea and pelvic pain. With advances in sonographic imaging, neonatal diagnosis is becoming more frequent and often occurs after the incidental identification of hydrocolpos or renal agenesis. On the other hand, prenatal diagnosis of OVHIRA is extremely rare, with only two reported cases in the literature to date. Han et al. described a case of prenatal diagnosis of OHVIRA at 37 weeks of gestation following visualization of an absent left kidney and a cystic mass in the retrovesical space [15]. Tuna et al. reported a case of prenatal diagnosis of OHVIRA at 36 weeks’ gestation after the detection of right renal agenesis and hydrocolpos. An MRI was performed in both cases to confirm the diagnoses [2]. These cases emphasize the significance of conducting a comprehensive prenatal sonographic examination to facilitate timely identification of OHVIRA syndrome during the prenatal phase. It is important to possess knowledge about OHVIRA syndrome for early diagnosis and subsequent treatment to prevent potential complications such as endometriosis, infertility, and spontaneous abortion. There is no clear optimal treatment for OHVIRA syndrome; however, most clinicians agree that, in most cases, resection of the vaginal septum restores reproductive function [10]. The resection is usually not an emergency and should be performed around the pubertal period, except for rare cases where OHVIRA is complicated by infections, such as pelvic inflammatory disease and abscess formation [11,13]. Surgical treatment is important and is the optimal treatment to relieve obstruction and symptoms, prevent complications of retrograde flow, and preserve fertility [14,16]. OHVIRA with a didelphys uterus has not been associated with infertility or pregnancy complications, such as spontaneous miscarriage or preterm delivery. However, OHVIRA with a septate uterus is a known risk factor for infertility and a cause of pregnancy complications, and pregnancy outcomes can be markedly improved after resection of the uterine septum [16]. The presence of a thick vaginal septum may restrict the distal distension of the affected hemivagina, leading to retrograde menstrual bleeding into the peritoneal cavity, ultimately resulting in endometriosis. Patients with OHVIRA have been reported to have a prevalence of endometriosis as high as 23%. Treatment with gonadotropin-releasing hormone analogs may be a good option for maintaining amenorrhea and reducing pelvic pain associated with endometriosis [10,11].

Gartner duct cysts are extremely rare in fetuses and neonates. They result from obstruction of the mesonephric duct system during fetal development and have been reported to be caused by a failure of separation of the ureteric bud from the mesonephric duct, but this remains unclear [5]. Gartner duct cysts are located in the anterior or lateral wall of the vagina. The vaginal cysts are lined with stratified squamous epithelium as they originate from the Müllerian duct, while Gartner duct cysts are lined with cuboidal epithelium. The majority of cases are asymptomatic and detected incidentally. The association of Gartner duct cysts with ipsilateral renal agenesis or dysplasia is infrequent and results from the abnormal development of the ureter [6]. For an accurate diagnosis, a physical examination and imaging tests, such as an ultrasound and an MRI, should be performed. During the examination, the patient should be positioned in the frog-leg position, and the labia majora should be delicately grasped and laterally pulled to examine the introitus and vagina. The management options are aspiration, deroofing, and the removal of the entire cyst through a vaginal approach [6]. The optimal timing for the treatment of Gartner duct cysts remains uncertain, particularly in newborns. Nevertheless, the long-term prognosis is generally favorable. 

## 4. Conclusions

It is common for individuals with OHVIRA syndrome to exhibit symptoms such as progressive dysmenorrhea, abnormal pain, menstrual irregularities, and pelvic masses after the onset of menarche. Therefore, it is recommended that appropriate medical attention and management, perhaps initiated during adolescence, be provided to individuals with OHVIRA syndrome in order to prevent complications such as endometriosis, infertility, and spontaneous abortion. OHVIRA syndrome is associated with a favorable prognosis, and severe complications can be avoided by early diagnosis and surgical intervention. Gartner duct cysts are rare, associated with renal anomalies, and have a favorable long-term prognosis. The novel aspect of this report is the presentation of a case accompanied by a Gartner duct cyst in addition to OHVIRA syndrome. Therefore, a differential diagnosis is crucial since other genitourinary anomalies, such as Gartner duct cysts, may coexist. Therefore, clinicians, especially obstetricians and pediatricians, should perform systematic ultrasound examinations to identify any other genitourinary anomalies when encountering female fetuses with ipsilateral renal agenesis or vaginal cysts.

## Data Availability

The data presented in this study are available on request from the corresponding author. The data are not publicly available due to privacy and ethical concerns.
